# Detection of Sarcopenia in Patients with Liver Cirrhosis Using the Bioelectrical Impedance Analysis

**DOI:** 10.3390/nu15153335

**Published:** 2023-07-27

**Authors:** Dorotea Bozic, Ivica Grgurevic, Bisera Mamic, Vesna Capkun, Josipa Bilandzic-Ivisic, Tomislav Ivanovic, Ivona Bozic, Ivan Zaja, Kristian Podrug, Zeljko Puljiz, Zdravko Perko, Ivana Mikolasevic

**Affiliations:** 1 Department of Gastroenterology, University Hospital of Split, Spinciceva 1, 21000 Split, Croatia; dora.bozic@hotmail.com (D.B.); josipa.bilandzic0000@gmail.com (J.B.-I.); ivan.zaja@yahoo.com (I.Z.); kpodrug@gmail.com (K.P.); zpuljiz4@gmail.com (Z.P.); 2 Department of Gastroenterology, Hepatology and Clinical Nutrition, Clinical Hospital Dubrava, Avenija Gojka Suska 6, 10000 Zagreb, Croatia; 3 School of Medicine, University of Zagreb, Salata 3, 10000 Zagreb, Croatia; 4 Faculty of Pharmacy and Biochemistry, University of Zagreb, Ante Kovacica 1, 10000 Zagreb, Croatia; 5 Department of Oncology and Radiotherapy, University Hospital of Split, Spinciceva 1, 21000 Split, Croatia; bisera208@gmail.com; 6 Department of Nuclear Medicine, University Hospital of Split, Spinciceva 1, 21000 Split, Croatia; vesna.capkun@gmail.com; 7 Department of Abdominal Surgery, University Hospital of Split, Spinciceva 1, 21000 Split, Croatia; tomo.mefst@gmail.com (T.I.); zperko@gmail.com (Z.P.); 8 Department of Rheumatology and Immunology, University Hospital of Split, Soltanska 2, 21000 Split, Croatia; ivona.bozic.7@gmail.com; 9 School of Medicine, University of Split, Soltanska 2, 21000 Split, Croatia; 10 Department of Oncology and Radiotherapy, University Hospital Center Rijeka, Kresimirova 42, 51000 Rijeka, Croatia; ivana.mikolasevic@gmail.com; 11 School of Medicine, University of Rijeka, Brace Branchetta 20/1, 51000 Rijeka, Croatia

**Keywords:** sarcopenia, liver cirrhosis, bioelectrical impedance analysis

## Abstract

Bioelectrical impedance analysis (BIA) is a body composition assessment method. We aimed to determine its accuracy in the detection of sarcopenia in patients with liver cirrhosis (LC), using skeletal muscle index (SMI) at the level of third lumbar vertebra (L3-SMI) obtained using multislice computed tomography as the reference method. Patients with LC were enrolled in the period October 2019–March 2022 and follow-ups were conducted until January 2023. Their BIA parameters were compared against L3-SMI, and BIA cut-off values were proposed using AUROC analysis. Patients underwent outcome analysis based on obtained clinical characteristics. A total of 106 patients were included. We found a fair correlation between BIA parameters with the L3-SMI. We determined cut-off values of ≤11.1 kg/m^2^ for BIA-SMI (Se 73%, Sp 66%, AUROC 0.737, *p* < 0.001) and ≤5.05° for phase angle (PA) (Se 79%, Sp 60%, AUROC 0.762, *p* < 0.001) in the detection of sarcopenia. The relative risk of death was 2.2 times higher in patients with skeletal muscle mass (SMM) ≤ 36.5 kg. SMM was significantly associated with outcome in Kaplan–Meier analysis. This non-invasive and simple method that showed fair performances and a very good outcome prediction could provide for the unmet need for fast and affordable detection of sarcopenia in patients with LC and should be further evaluated.

## 1. Introduction

Liver cirrhosis (LC) is an end stage liver disease, characterized by diffuse nodular regeneration of liver parenchyma surrounded by fibrotic septa and the consequent distortion of vascular architecture [1]. Usually caused by excessive alcohol consumption, non-alcoholic fatty liver disease (NAFLD) and chronic hepatitis B and C infection, LC presents an increasing cause of mortality in more developed countries [1]. Dense fibrotic liver parenchyma causes resistance to portal blood flow and leads to portal hypertension, which is an anchor point for disease decompensation that may manifest as ascites, variceal bleeding or hepatic encephalopathy [2]. The evolvement from compensated to decompensated disease is accompanied by an increase in the percentage of malnutrition from 20% in patients with compensated disease to 50% in patients with decompensated disease [3]. A recently published meta-analysis that included 8821 patients with LC found the pooled prevalence of sarcopenia to be 33% (95% CI 0.32–0.34) [4].

Sarcopenia, which is defined as generalized loss of muscle mass and function, is a major component of malnutrition [3]. Emphasizing its importance, it has the highest annual prevalence among all LC complications besides portal encephalopathy, with which it is assumed to have a causative relationship [5,6,7]. Sarcopenia develops as a result of several metabolic derangements occurring in patients with LC such as inadequate nutrition, systemic inflammation, inhibition of muscle growth due to high myostatin levels and excessive use of proteins as an energy resource [5].

Mechanisms participating in the development of malnutrition include excessive use of alcohol as energy resource, low appetite caused by salt and protein restrictions, nausea, dysgeusia, increased intra-abdominal pressure and the low socioeconomic status of some patients. Poor absorption, increased gastrointestinal permeability and bacterial overgrowth potentiate this condition [3,8,9]. The main metabolic alteration is a reduction in hepatic glycogen stores, leading to the use of body fat and protein stores for gluconeogenesis [5]. This process occurs even in the short periods of starvation, e.g., overnight. Since maintenance of quality and mass of skeletal muscle is essential for metabolic function and daily physical activity, sarcopenia negatively affects the flow of the disease, quality of life and survival, as well as patient outcomes after orthotopic liver transplantation (OLT) [5,8,9]. Therefore, the presence of sarcopenia should be assessed in all patients with LC to determine risk of poor outcomes, which is adopted as a recommendation in the European Association for the Study of the Liver (EASL), the American Association for the Study of Liver Diseases (AASLD) and the European Society for Clinical Nutrition and Metabolism (ESPEN) clinical practice guidelines [3,10,11].

Several methods have been developed for sarcopenia assessment, including the mid-arm muscle circumference (MAMC), muscle function tests (hand grip strength (HGS) and short physical performance battery (SPPB)), dual energy X ray absorptiometry (DEXA), multislice computed tomography (MSCT), magnetic resonance imaging (MRI) and bioelectrical impedance analysis (BIA). Anthropometric measurements have high interobserver variability and are not able to distinguish between lean and fat mass, which is of particular relevance considering the increasing rates of obesity in patients with LC [10]. On the contrary, an MSCT scan differentiates fat from other soft tissues, allowing for accurate estimation of the fat and skeletal muscle mass, while the cross-sectional area at the third lumbar vertebra correlates well with the whole body muscle mass [12]. Therefore, determining the skeletal muscle index (SMI) at the level of the third lumbar vertebra using the abdominal MSCT scan (L3-SMI), has been recognized and adopted in clinical guidelines as the gold standard in diagnosis of sarcopenia [8,10,11,13,14,15,16,17]. Although abdominal MSCT scan is commonly used in cirrhotic patients for diagnosis and staging of hepatocellular carcinoma or to ascertain splanchnic vein thrombosis, its use is not appropriate merely for the detection of sarcopenia due to radiation exposure, high cost, possible contrast-induced kidney injury or precipitation of hepatorenal syndrome [10]. Taking in mind these drawbacks, MSCT would also not be a reasonable method for the monitoring of sarcopenia, considering the need for its reassessment at least annually for patients with compensated LC and every 3 months for patients with decompensated LC or patients under active treatment [10]. Additionally, the process of L3-SMI measurement is time consuming and requires training. On the other hand, BIA is a portable, swift, low cost and easy-to-use modality with no radiation exposure.

Hence, we aimed to determine the performance of BIA in detection of sarcopenia in patients with LC, using L3-SMI as a reference method, and to analyze interrelation of patient’s clinical characteristics and outcomes with the presence of sarcopenia.

## 2. Materials and Methods

This prospective observational study was conducted at the Department of Gastroenterology, University Hospital of Split. Patients with LC that were treated as in-hospital or out-hospital patients, regardless of etiology of the liver disease, were enrolled from October 2019 until March 2022. The last day of follow-up was the date of death from any cause for deceased patients and the day of OLT for transplanted patients, while 31 December 2022 was the last date of clinical follow up for patients who survived and were not transplanted.

Adult patients of both genders, with compensated (Child-Turcotte-Pugh (CTP) A) or decompensated (CTP B and C) LC, without exclusion criteria, were included in the study after signing the informed consent form. The diagnosis of LC was established on the grounds of physical examination, laboratory tests and the multiparametric abdominal ultrasound.

Exclusion criteria were as follows: presence of hepatocellular cancer or other malignant disease, previous OLT, active alcohol abuse, HIV positivity, acute liver, kidney, cardiac or pulmonary failure and sepsis. Patients with overt hepatic encephalopathy or who were not able to provide informed consent due to other neurological/psychiatric disorders were not included in the study. Patients with an amputated limb, orthopedic prosthesis use, cardiac pacemaker or implantable cardioverter defibrillator (ICD) were not included in the study.

The data on patient’s gender, age, body mass, height, body mass index (BMI), etiology of the disease (ethylic, NAFLD, hepatitis B, hepatitis C and other), previous LC complications (portal encephalopathy, variceal bleeding and ascites), use of diuretics, grade of ascites (none, mild, moderate and severe) and the peripheral edema (none, mild, moderate and severe) were collected, as well as the laboratory parameters including total bilirubin, creatinine, international normalized ratio (INR), albumins, platelets, natrium and potassium. CTP score, model for end stage liver disease (MELD) score and MELD-sarcopenia score were calculated.

The MELD-sarcopenia score was calculated using the formula: MELD + (10.35 × sarcopenia), where sarcopenia was replaced with zero (0) in the absence of sarcopenia and with one (1) when sarcopenia was present. Since Montano-Loza introduced this formula and based the definition of sarcopenia on following SMI cut-off values (males with BMI < 25: <43 cm^2^/m^2^, males with BMI > 25: <53 cm^2^/m^2^, females: <41 cm^2^/m^2^), we defined sarcopenia using aforementioned cut-off values only for the purposes of calculating this score [18]. Adverse outcome was defined as death or OLT.

**Figure 1:**
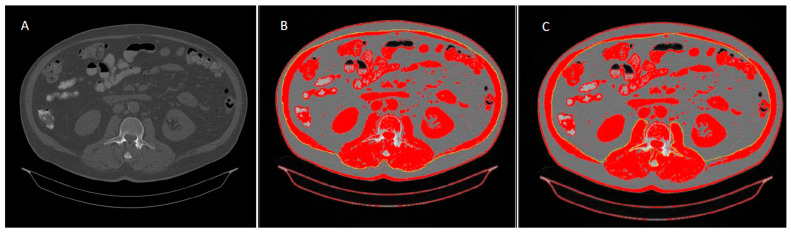
L3-SMI measurement: transverse scan at the level of the L3 vertebra obtained by the MSCT (**A**) which is then imported into the ImageJ software to delineate the abdominal musculature from outer (**B**) and the inner (**C**) side. L3-SMI: skeletal muscle index at the level of the third lumbar vertebra; MSCT: multislice computed tomography; L3: third lumbar vertebra.

Abdominal MSCT scans were analyzed by a trained radiotherapist. A transverse CT scan at the level of L3 vertebra was incorporated into the National Institutes of Health ImageJ software version 1.48 for each patient and the L3-SMI analysis was performed. Skeletal muscles, including psoas, quadrantus lumborum, transversus abdominus, erector spinae, external and internal obliques and rectus abdominis, were identified and demarcated using Hounsfield unit (HU) thresholds from −29 to +150 (Figure 1). Cross-sectional areas (cm^2^) were then measured and normalized for height (m^2^) to calculate the L3-SMI. Patients fulfilling recently proposed Carey’s cut-off values (≤50 cm^2^/m^2^ for men and ≤39 cm^2^/m^2^ for women) were defined as sarcopenic [14].

BIA was performed within a week after the MSCT scan. After fulfilling the procedure preparation demands, BIA analysis was conducted on the multi-frequency segmental BIA analyzer Tanita MC-780MA (Tanita Corporation, Tokyo, Japan) by a trained nurse with experience in this procedure [19]. This multifrequency BIA analyzer uses 90 A current with three different frequencies (5, 50, 250 kHz), and provides both segmental and whole body muscle and fat mass analysis [20]. During measurement, the patient stands with bare feet on the metal foot plate, gently holding the hand grip in neutral standing position. Measurement lasts for less than 20 s. The instrument consists of eight pairs of tactile electrodes incorporated into the stainless steel foot pad and plated handgrips (two electrodes for each foot/hand). After sending a low electrical signal to active tactile electrodes, the system measures the resistance and reactance between two other passive tactile electrodes, which is known as the tetra-polar mode. BIA software MC-780 MA determines total body water (TBW), extracellular water (ECW), intracellular water (ICW), fat mass (FM), fat free mass (FFM), skeletal muscle mass (SMM) and phase angle (PA). Inbuilt software measures SMM using the Jansenn equation, and calculates the phase angle (PA), which is measured at the frequency of 50 kHz, using the following formula: phase angle (PA): Reactance (Xc)/Resistance (R) × (180/π) [21]. SMM was normalized for height (m^2^) to calculate the BIA-SMI.

All collected data were organized in excel sheets and statistically analyzed. The study was conducted in accordance with the provisions of the Declaration of Helsinki and was approved by the ethics committee of the University Hospital Split (500-03/19-01/74).

Statistical Package for the Social Sciences (SPSS) software (version 20 for Windows; SPSS Inc., Chicago, IL, USA) was used in statistical analysis. The categorical variables were presented as count and frequency, the continuous quantitative variables were presented as mean ± standard deviation (95% confidence interval (CI)) and the noncontinuous quantitative variables were presented as median (interquartile range; min–max). For normally distributed continuous variables a *t*-test was used, and for abnormally distributed continuous variables, a nonparametric Mann–Whitney U test was applied. An χ^2^ test was used for the comparison of categorical variables. Correlation was assessed using the Spearman correlation coefficient. BIA parameters as predictors for sarcopenia were estimated using the univariate and multivariate logistic regression analysis, with odds ratio (OR), 95% CI and *p*-value reported. ROC curve analysis was used to evaluate BIA performance and to determine cut-off values. Log-rank test and the Kaplan–Meier curves were used for the outcome analyses. The significance level was determined as *p* < 0.05.

## 3. Results

### 3.1. Baseline Patient Characteristics

A total of 106 patients were included in the study. There were 16 (15%) female and 84 male (85%) participants. The mean age was 59 ± 9.5 years (57–61), without a significant difference between the genders (t = 0.344, *p* = 0.731). Most of the patients had alcohol-related (85%) and decompensated (79%) liver cirrhosis. The patients’ demographic and clinical characteristics and laboratory data, as well as the mean values of BIA and MSCT parameters, are systematically presented in Table 1.

### 3.2. Correlation between BIA Parameters and the L3-SMI

After dividing patients into groups according to their volume status, we calculated the Spearman correlation coefficients and found the strongest correlations of SMM, SMI and FFM with the L3-SMI in the group without or with mild ascites/peripheral edema (Table 2). The correlation in the aforementioned group was fair (R = 0.498 and R = 0.434) for SMM and FFM, respectively, and moderate for the SMI (R = 0.614). Regarding the PA, we found a fair correlation between the methods in the whole cohort (R = 0.571) and in the group of patients without or with mild ascites/peripheral edema (R = 0.524). This correlation, however, was shown to be moderate in the group of patients with moderate or severe ascites/peripheral edema (R = 0.676) (Table 2).

### 3.3. Patient Characteristics Regarding the Presence of Sarcopenia

According to the L3-SMI values, a total of 56 patients (53%) were classified as sarcopenic using the Carey criteria. The distribution of patients according to gender did not differ significantly in relation to sarcopenia (X^2^ = 0.277; *p* = 0.599). We also did not prove a statistically significant difference regarding the age of patients in relation to sarcopenia (t = 1.66; *p* = 0.100). We found significantly higher values of SMM, SMI, FFM and PA in patients without sarcopenia than in the group of patients with sarcopenia (t = 3.33, *p* = 0.001; t = 4.4, *p* < 0.001; t = 2.9, *p* = 0.005 and t = 5.2, *p* < 0.001, respectively). Distribution of patients regarding gender, age and BIA parameters in relation to sarcopenia is presented in Table 3.

### 3.4. BIA Performance and Cut-Off Values in Detection of Sarcopenia

**Figure 2:**
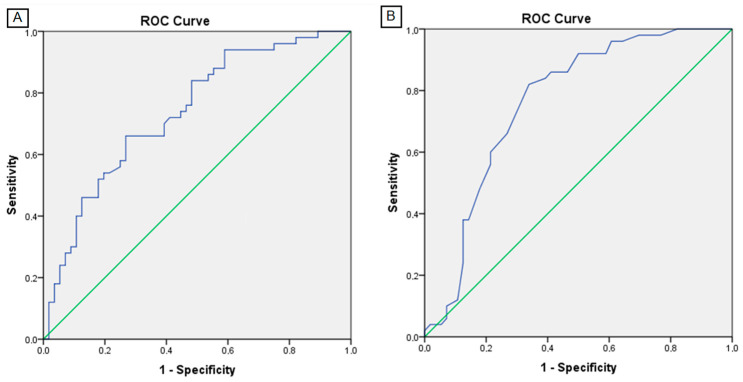
BIA performance in detection of sarcopenia using ROC curve analysis. ROC curves demonstrating the detection of sarcopenia using BIA parameters: BIA-SMI (**A**) and PA (**B**); the corresponding AUROCs were 0.737 and 0.762, respectively. Green line: random chance, blue line: predictive model. ROC: receiver operating characteristic; AUROC: area under the ROC curve.

Using the L3-SMI as the reference method, we analyzed the performance of BIA parameters in the detection of sarcopenia using ROC-curve analysis (Table 4). We determined a cut-off value of 11.1 kg/m^2^ for BIA-SMI (sensitivity (Se) 73%, specificity (Sp) 66%, area under the ROC curve (AUROC) 0.737, 95% CI (0.643–0.831), *p* < 0.001) and cut-off value of 5.05° for the PA (Se 79%, Sp 60%, AUROC 0.76, 95% CI (0.669–0.855), *p* < 0.001). Figure 2 presents ROC curves of BIA-SMI (A) and PA (B) for detection of sarcopenia in patients with LC.

After dividing patients into two groups according to the previously obtained cut-off values of the BIA parameters, we analyzed their association with the sarcopenia (L3-SMI) and performed univariate logistic regression analysis. Using the proposed BIA SMI cut-off values, the percentage of patients with sarcopenia in our cohort was 39%. We found that the percentage of patients with the BIA-SMI ≤ 11.1 kg/m^2^ was 2.2. times higher in the group of patients with sarcopenia than in patients without sarcopenia (X^2^ = 14.9; *p* < 0.001). The probability of occurrence of sarcopenia in the group of subjects with the BIA-SMI ≤ 11.1 kg/m^2^ was 5.3 times higher than the probability of occurrence in the group of BIA-SMI > 11.1 kg/m^2^ (OR 5.3, 95% CI (2.3–12), *p* < 0.001). The results for all tested variables are systematically presented in Table 5.

We performed the multivariate logistic regression analysis using BIA parameters and found that the SMI and PA are statistically significantly associated with the occurrence of sarcopenia (*p* = 0.002 and <0.001, respectively). In extension, we performed the multivariate logistic regression analysis for occurrence of sarcopenia using only the two aforementioned independent variables (Table 6). We found that the probability of occurrence of sarcopenia in the group of subjects with the SMI ≤ 11.1 kg/m^2^ is 4.5 times higher than in the group of subjects with SMI > 11.1 kg/m^2^ (*p* = 0.001), and the probability of occurrence of sarcopenia in the group of subjects with PA ≤ 5.05° is 4.7 times higher than in the group of subjects with PA > 5.05° (*p* = 0.001).

Additionally, we determined dichotomized cut-off values optimized for sensitivity and specificity to determine rule-in and rule-out criteria for SMI and obtained the following values: SMI ≤ 9.9 kg/m^2^ to rule-in sarcopenia (Sp 88%, 95% CI (64–100), Se 41%, 95% CI (32–75), positive predictive value (PPV) 79%, 95% CI (50–100), negative predictive value (NPV) 57%, 95% CI (41–77); *p* = 0.002) and SMI > 13.02 kg/m^2^ to rule-out sarcopenia (Se 91%, 95% CI (68–100), Sp 30%, 95% CI (17–49), PPV 59%, 95% CI (44–78) and NPV 75%, 95% CI (42–100), *p* < 0.012).

**Scheme 1:**
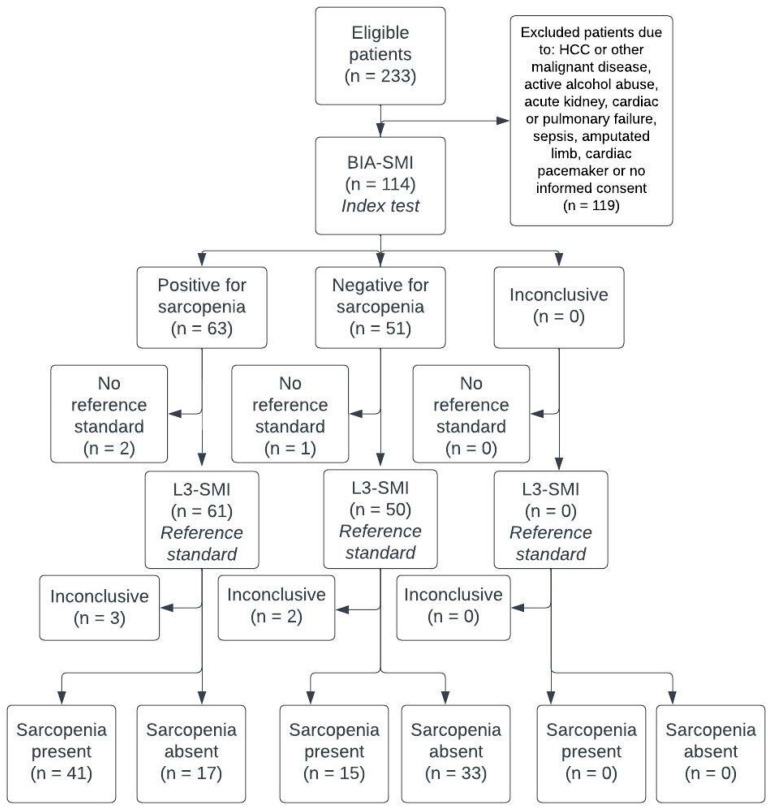
STARD flowchart presents the diagnostic accuracy of the BIA-SMI in detection of sarcopenia in patients with LC. STARD: standards for reporting diagnostic accuracy studies; HCC: hepatocellular cancer.

Finally, the standards for reporting diagnostic accuracy studies (STARD) flowchart is presented to demonstrate the diagnostic accuracy of the BIA-SMI in the detection of sarcopenia in patients with LC (Scheme 1).

The median MELD score was 15 (Q1–Q3: 11–19; min–max: 7–42). We did not find a statistically significant difference regarding MELD score between subjects with and without sarcopenia (Z = 0.415; *p* = 0.678). The median MELD-sarcopenia score in the group without sarcopenia was 18 (Q1–Q3: 12–24; min–max: 7–42), and in the group with sarcopenia 24 (Q1–Q3: 20–29; min–max: 8–43). There was a statistically significant difference in the MELD-sarcopenia scores between the groups without and with sarcopenia (Z = 4.0; *p* < 0.001).

### 3.5. Outcome Analysis

As previously stated, adverse outcome was defined as death or OLT. The average overall survival without OLT was 24.3 months (SE: 1.5, 95% CI 21–27).

We divided patients into three groups based on their MELD score: group 1: MELD ≤ 10, group 2: MELD 11–19 and group 3: MELD ≥ 20. Groups 1 and 2 did not differ significantly in terms of outcome, therefore we combined these two groups, compared them with group 3 and obtained a statistically significant difference: the relative risk of adverse outcome in group 3 was 1.5 times higher (HR 1.5, 95% CI (1.1–2), *p* = 0.017) than in groups 1 and 2 combined. We also divided patients regarding MELD-sarcopenia score into two groups (MELD-sarcopenia ≤ 27 and >27) and found that the relative risk of adverse outcome was 1.4 times higher (HR:1.4, 95% CI (1.1–1.7), *p* = 0.002) in the group with MELD-sarcopenia score ≤ 27. Similarly, we combined CTP groups A and B and compared their outcome with the CTP C group, finding a statistically significant difference: the relative risk of adverse outcome in the CTP C group was 1.5 times higher (HR 1.5, 95% CI (1.1–2.1), *p* = 0.006) than in groups A and B combined.

We found that the risk of adverse outcome increased by 1.06 (HR:1.06, 95% CI (1–1.1), *p* = 0.002) for each unit increase in MELD-sarcopenia score, and likewise for MELD (HR = 1.12, 95% CI (1.1–1.2), *p* < 0.001) and CTP scores (HR = 1.7, 95% CI (1.1–2.7), *p* = 0.011).

We also determined a relative risk of adverse outcome being 2.2 times higher (HR 2.2, 95% CI (1.2–4.0), *p* = 0.015) in the group of patients with the BIA-SMM ≤ 36.5 kg than in the group with the BIA-SMM > 36.5 kg, and 2 times higher (HR 2, 95% CI (1.1–3.8), *p* = 0.021), in the group with the FFM ≤ 66.8 kg than in the group with the FFM > 66.8 kg.

**Figure 3:**
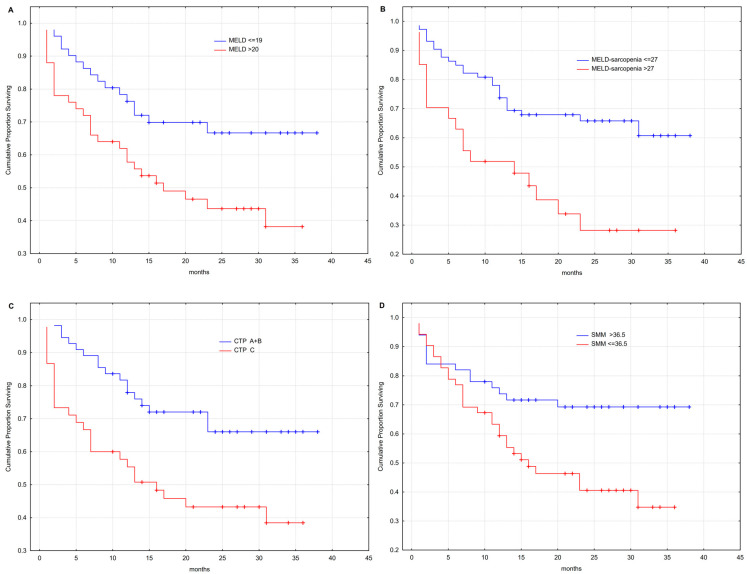
Kaplan–Meier survival analysis. Kaplan–Meier estimates for the probability of overall survival without OLT in patients according to: MELD score (**A**), MELD-sarcopenia score (**B**), CTP score (**C**) and BIA-SMM (**D**). OLT: orthotopic liver transplantation.

MELD, MELD-sarcopenia and CTP scores, as well as the BIA-SMM were significantly associated with outcome in Kaplan–Meier analysis (Figure 3).

## 4. Discussion

In this prospective study we demonstrated a fair correlation of BIA parameters with the L3-SMI as a reference method. Additionally, we proposed SMI and PA cut-off values for detection of sarcopenia in Caucasian patients with LC and proved the deleterious impact of muscle mass loss on patient outcome.

An exemplary method for the detection of sarcopenia should be accurate, but also accessible, highly reproducible, affordable and safe, and be able to predict significant endpoints such as mortality [22]. There are several advantages of BIA over the MSCT in evaluation of sarcopenia, that could simplify and precipitate its diagnosis in daily clinical practice. BIA is an inexpensive, portable, non-radiating, easily available method that does not preclude contrast application. Its use may be repeated enabling the patient follow-up, which is of significant importance in dietary and exercise interventions. However, it has its limitations in immobile and severely ill patients, individuals with limb amputation or orthopedic prothesis, or with an ICD or cardiac pacemaker. Moreover, the accuracy of BIA in patients with moderate or severe ascites/peripheral edema has been the matter of debate between the experts, since this controversy is of particular importance in patients with LC.

According to the Japan Society of Hepatology, ascites does not have a major impact on the assessment of sarcopenia. This conclusion is based on a study that included 149 patients with LC, in which authors found a strong correlation (R = 0.72) between the BIA-SMI and the L3-SMI, irrespective of the presence of ascites [23]. Contrary, when Woodward AJ et al. compared several bedside techniques in the assessment of muscle mass, bioelectrical impedance spectroscopy (BIS) showed the strongest correlation coefficients (r = 0.78–0.79; *p* < 0.01), although weaker correlations were found in patients with ascites for all methods, except when using the MUAMC [24]. Several authors reported that measuring the arm index (SMM of arms divided by squared height) might be useful for avoiding the effects of severe edema [25,26]. In 2022, a Thai study including 30 cirrhotic patients with sarcopenia (determined by HGS) evaluated the utility of BIA-SMI in comparison to the MSCT as the reference [27]. Only patients with < grade 2 ascites were included in the study, and the authors found a fair correlation between the methods (r = 0.54; *p* < 0.002) [27]. We also found a fair correlation (R = 0.509) between the BIA-SMI and the L3-SMI in the complete cohort of patients, but that correlation was moderate (R = 0.614) in the group of patients without or with mild ascites/peripheral edema, indicating the potential influence of the fluid overload on the SMI measurement (Table 2).

Apart from analyzing the SMI, we also studied the PA, which is an indicator of cell membrane integrity and functionality. Since the higher PA stands for preserved cellular integrity and represents the higher muscle mass, it may be used as indicator for sarcopenia [28]. We found a fair correlation between the PA and the L3-SMI (R = 0.571, *p* < 0.001) in the complete patient cohort, and, unexpectedly, moderate correlation (R = 0.676, *p* < 0.001) in the group of patients with moderate or severe ascites/ peripheral edema. Ruiz-Margáin A. et al. also disproved influence of ascites on the estimation of PA in a study that included 136 patients with LC: authors found a fair correlation of PA with the L3 SMI (r = 0.58, *p* < 0.001), irrespective of the ascites presence [28].

The correlation of BIA parameters with the L3-SMI has also been studied in other patient groups, including critically ill and patients with colorectal cancer (CRC) [12,29]. Kim E.Y. et al. evaluated accuracy of multifrequency BIA in 50 patients with CRC and found a strong correlation (r = 0.705, *p <* 0.001) between the BIA-SMM and L3-SMI, that remained significant (r = 0.641, *p* < 0.001) after adjusting for age, weight and the BMI values [12]. Kim D. et al. evaluated accuracy of BIA on 135 critically ill surgical patients and found that the correlation was dependent on the sex of the patient and the severity of edema: the correlation was stronger in the male group (r = 0.651) and in patients who had mild edema (r = 0.734), than in the female group (r = 0.584) or patients with severe edema (r = 0.613) [29]. However, the highest published correlation between the BIA-SMM and the MSCT-SMM was determined in the study that included 1191 healthy subjects who were evaluated at the health promotion center in Korea (r = 0.898, *p* < 0.001) [30].

As Sinclair M. stated, little work has been conducted to validate accuracy of BIA in patients with LC [22]. Compared to a significant number of Asian studies that assessed BIA in detection of sarcopenia in patients with LC, studies performed on Caucasian examinees are lacking, have proposed cut-off values only for the PA or did not use L3-SMI as the reference method. Therefore, we used ROC analysis to propose first cut-off values for BIA parameters in detection of sarcopenia in Caucasian patients with LC, which is the main contribution of our study. We determined cut-off value of 11.1 kg/m^2^ (Se 73%, Sp 66%, AUROC 0.737, 95% CI (0.643–0.831); *p* < 0.001) for the BIA-SMI. After performing the multivariate logistic regression analysis, we found that the probability of occurrence of sarcopenia in the group of subjects with the BIA-SMI ≤ 11.1 kg/m^2^ is 4.5 times higher than in the group of subjects with BIA-SMI > 11.1 kg/m^2^ (*p* = 0.001). Additionally, we obtained SMI dichotomized threshold values optimized for sensitivity and specificity (SMI ≤ 9.9 kg/m^2^ as rule-in and >13.02 kg/m^2^ as rule-out criteria) to categorize certain patients with a higher degree of reliability into groups with or without sarcopenia.

We also determined the cut-off value for PA of 5.05° (Se 79%, Sp 60%, AUROC 0.762, 95% CI (0.669–0.855), *p* < 0.001) and found that the probability of occurrence of sarcopenia in the group of subjects with PA ≤ 5.05° is 4.7 times higher than in the group of subjects with PA > 5.05° (*p* = 0.001) in the multivariate logistic regression analysis. In 2017, a group of authors assessed the accuracy of PA in diagnosis of sarcopenia (determined with DEXA and HGS) on a cohort of 122 male patients with LC. Interestingly, authors found the same PA value (≤5.05°) to predict the diagnosis of sarcopenia with high sensitivity [31].

In 2015, Ruiz-Margáin A. et al. proposed a PA cut-off value of ≤4.9° which was obtained from a pilot study using the ROC analysis [32]. In 2017, Belarmino G. et al. evaluated this cut-off value on a cohort of 134 male patients with LC and found that it significantly and independently affected the mortality [33]. In 2021, Ruiz-Margáin A. et al. proposed new PA cut-off values in their cohort of 136 patients with LC (≤5.6° for males and ≤5.4° for females), using the L3-SMI as the reference method [28]. Several other authors also proposed various PA cut-off values (≤5.1–5.52°) in patients with decompensated liver cirrhosis [34,35]. Hernández-Conde M. et al. developed a high performance (AUROC 0.8) nomogram based on gender, BMI and PA to rule out the presence of sarcopenia in cirrhotic patients [36].

Various studies have evaluated the prevalence of sarcopenia in patients with LC, it’s influence on the quality of life, waiting time on the transplant list and the outcomes. In our predominantly male (85%) cohort, 82% of patients had ethylic etiology of LC, with the high rate of decompensated liver disease according to CTP (78.8%), and MELD score (Mdn 15, Q1–Q3: 11–19). According to the L3-SMI based on Carey criteria, a total of 56 patients (53%) were classified as sarcopenic, and when using the BIA-SMI determined cut-off value of ≤11.1 kg/m^2^, this percentage decreased to 39%. These results are similar to prevalence reports from other studies ranging from 37.5% to 57% [13,37,38,39].

In 2018, van Vugt J.L.A. and co-authors evaluated the MELD-Sarcopenia score which was initially established by Montano-Loza et al. [18,38]. Authors included 585 patients that were awaiting liver transplantation, among which 43.4% were sarcopenic. Patients with sarcopenia had shorter median waiting list survival (*p* < 0.001), but the authors did not find the additional MELD-sarcopenia score value in predicting waiting list mortality [38]. In our cohort, patients with MELD-sarcopenia score > 27 had 1.4 times higher (HR: 1.4, 95% CI (1.1–1.7), *p* = 0.002) risk of fatal outcome compared to patients with MELD-sarcopenia score ≤ 27. Similarly, patients with MELD score ≥ 20 and CTP grade C had 1.5 (HR 1.5, 95% CI (1.1–2), *p* = 0.017) and 1.5 times (HR 1.5, 95% CI (1.1–2.1), *p* = 0.006) higher relative risk of fatal outcome than patients with MELD < 20 or patients in groups CTP A and B, respectively. To emphasize, we also did not find any additional value of MELD-sarcopenia score over the MELD score, since the attributed hazard ratio was similar.

In our cohort of 106 patients with LC, we proved a 2.2 times higher risk of mortality (HR 2.2, 95% CI (1.2–4.0), *p* = 0.015) in the group of patients with the lower muscle mass (BIA-SMM ≤ 36.5 kg) and a two times higher mortality risk (HR 2.0, 95% CI (1.1–3.8), *p* = 0.021) in the group with the lower fat free mass (FFM ≤ 66.8 kg), thereby confirming the well-known impact of the skeletal muscle mass on the patient survival. BIA-SMM also demonstrated an unfavorable effect on long-term survival in the Kaplan–Meier analysis (Figure 3). Tantai X. et al. published a large meta-analysis that included 6965 patients with LC, among whom 37.5% were sarcopenic. Sarcopenia was independently associated with a twofold higher risk of mortality (HR 2.30, 95% CI 2.01–2.63), and the mortality rate increased with greater severity or longer duration of sarcopenia [37]. Another meta-analysis, published by Kim G. et al. in 2017. included 4037 patients from 20 studies (7 included Asian and 13 Western participants), among whom 48% were sarcopenic. Only two studies used BIA, and the other used MSCT based SMI or total psoas muscle area. The group with the sarcopenia had 1.72 times higher mortality compared to the non-sarcopenic group (HR 1.72, 95% CI (1.27–2.32), *p < 0*.001) [13].

Cho Y.S. et al. demonstrated on cohort of 160 patients with LC that MELD score, hepatic venous pressure gradient (HVPG) and sarcopenia are independently associated with the long-term mortality with following hazard ratios: 1.086, 1.076 and 1.890, respectively [40]. Topan M.M. and co-authors also demonstrated increased risk for ascites (OR 3.78, 95% CI (0.85–16.86), *p* = 0.049), hepatocellular carcinoma (OR 9.23, 95% CI (2.42–35.16), *p* = 0.0001), urinary tract infection (OR 4.83, 95% CI (1.77–13.22), *p* = 0.002) and spontaneous bacterial peritonitis (OR 2.49, 95% CI (0.63–9.77), *p* = 0.03) in sarcopenic patients with LC. In their cohort of 201 patients, apart from reduced 6-months and 12-months survival, sarcopenia was also associated with the more extended hospital stay and higher 30-day readmission rates (*p* < 0.0001) [39].

Considering a high prevalence rate and the evident deleterious impacts on the disease course and patient survival, sarcopenia is a serious and underestimated condition that should be evaluated in all patients with LC. From the diagnostic aspect, BIA could offer the unmet need for its simple, fast and non-costly detection.

This study has several limitations. Our cohort included a small percentage of female patients; hence the separate analysis and gender related cut-off values could not be established. In addition, significant proportion of patients had moderate or severe ascites/peripheral edema which could influence the BIA analysis.

## 5. Conclusions

To conclude, the performances of BIA are fair based on the results of our research. However, considering it is a non-invasive and simple method that showed a very good outcome prediction, its use could have a beneficial value in the clinical practice. Additionally, use of the dichotomized threshold values optimized for sensitivity and specificity may categorize certain patients with a higher degree of reliability into groups with and without sarcopenia. Multicentric studies with a larger number of participants are warranted for its further validation. Prevention, early recognition and treatment of sarcopenia in order to change the disease course and the patient outcome are of the utmost importance.

## Figures and Tables

**Table 1:** Baseline characteristics of the studied cohort of patients.

Parameter	Value	*p*
**Demographic characteristics**		
**Gender, *n* (%)**		
Female/Male	16/84 (15/85)	
**Age (AM ± SD [95% CI])**		
Total	59 ± 9.5 (57–61) y	
Female	59.3 ± 8.9 y	0.731 *
Male	58.4 ± 12.6 y	
**Body mass (AM ± SD)**		
Female	65.8 ± 14 kg	
Male	91 ± 17 kg	
**Body height (AM ± SD)**		
Female	166 ± 4.2 cm	
Male	179.8 ± 7.3 cm	
**BMI (AM ± SD [95% CI])**		
Total	27.5 ± 5.3 (26.4–28.5) kg/m^2^	
Female	23.8 ± 4.8 (21–26) kg/m^2^	0.002 *
Male	28 ± 5.1 (27–29) kg/m^2^	
**Clinical characteristics**		
**Etiology, *n* (%)** **^ † ^**		
Ethylic	87 (83)	
Chronic HBV infection	3 (3)	
Chronic HCV infection	11 (10.5)	
NAFLD	4 (3.8)	
Other	8 (7.6)	
**Prev.decomp, *n* (%)** **^ † ^**		
None	31 (30)	
Ascites	55 (53)	
Portal encephalopathy	18 (17)	
Variceal bleeding	18 (17)	
**CTP score, *n* (%)**		
A	22 (21.2)	
B	36 (34.6)	
C	46 (44.2)	
**Ascites grade, *n* (%)**		
0	32 (30.8)	
1	25 (24)	
2	18 (17.3)	
3	29 (27.9)	
**Peripheral edema grade, *n* (%)**		
0	60 (57.1)	
1	21 (20)	
2	21 (20)	
3	3 (2.9)	
**Diuretics, *n* (%)**	61 (58)	
**Laboratory values ** (Mdn (Q1–Q3; min–max))		
Platelets (×10^9^/L)	110.5 (74.5–151; 26–299)	
INR	1.47 (1.28–1.83; 0.61–4.5)	
Albumins (g/L)	30.3 (26–35; 18–48)	
Bilirubin (µmol/L)	45 (26.5–92; 6–457)	
Creatinine (µmol/L)	69 (59–85; 27–305)	
Natrium (mmol/L)	136.5 (134–139; 117–144)	
Potassium (mmol/L)	4 (3.7–4.4; 2–6)	
MELD score	15 (11–19; 7–42)	
MELD-sarcopenia score	22 (15–27; 7–43)	
**MSCT and BIA parameters** (AM ± SD [95% CI])		
**MSCT**		
L3 surface (cm^2^)	152 ± 32 (146–159)	
Body height (cm)	178 ± 8.7 (176–179)	
L3 SMI (cm^2^/m^2^)	47.9 ± 8.9 (46.3–49.7)	
**BIA**		
SMM (kg)	35.8 ± 9 (34–37)	
SMI (kg/m^2^)	11.3 ± 2.4 (10.8–11.7)	
ECW (kg)	19.9 ± 3.6 (19–21)	
ICW (kg)	26.9 ± 6.7 (25.6–28)	
TBW (kg)	47.2 ± 10.1 (45.3–49)	
FFM (kg)	66 ± 12.8 (63.6–68.4)	
PA (°)	4.8 ± 0.89 (4.6–5)	

* *t*-test for independent variables. ^†^ Certain patients had several disease etiologies or manifestations of previous decompensation; therefore, the sum is not equal to the total number of patients. AM: arythmetic mean; SD: standard deviation; 95% CI: 95% confidence interval; y: year; BMI: body mass index; HBV: hepatitis B virus; HCV: hepatitis C virus; NAFLD: non-alcoholic fatty liver disease; Prev.decomp: previous decompensations; CTP: Child-Turcotte-Pugh; Mdn: median; Q1–Q3: interquartile range; min–max: minimum–maximum; INR: international ratio; MELD: model for end stage liver disease; BIA: bioelectrical impedance analysis; SMM: skeletal muscle mass; SMI: skeletal muscle index; ECW: extracellular water; ICW: intracellular water; TBW: total body water; FFM: fat free mass; PA: phase angle.

**Table 2:** Correlation between L3 SMI and BIA parameters.

	R (*p*)		
	Total	Ascites/Peripheral Edema	
		Grade 0 and 1	Grade 2 and 3
SMM	0.443 (<0.001)	0.498 (<0.001)	0.390 (0.007)
SMI	0.525 (<0.001)	0.614 (<0.001)	0.470 (0.001)
FFM	0.404 (<0.001)	0.434 (0.003)	0.418 (0.004)
PA	0.571 (<0.001)	0.524 (<0.001)	0.676 (<0.001)

R: Spearman correlation coefficient.

**Table 3:** Distribution of gender, age and BIA parameters of the studied cohort in relation to sarcopenia defined by L3-SMI using Carey criteria.

	Sarcopenia (L3-SMI)		*p*
	No (*n* = 49)	Yes (*n* = 56)	
Gender; *n* (%)			0.599 *
Male	43 (88)	46 (82)	
Female	6 (12)	10 (18)	
Age (years)			
(AM ± SD (min–max))	57.6 ± 9.3 (36–77)	60.4 ± 9.6 (43–85)	0.100 **
BIA variables (AM ± SD (min–max))			
SMM (kg)	38.8 ± 8.8 (23–56)	33.2 ± 8.4 (17.4–57.4)	0.001 **
SMI (kg/m^2^)	12.3 ± 2.3 (8.4–17.3)	10.4 ± 2.1 (6.7–18)	<0.001 **
FFM (kg)	69.6 ± 12 (43.6–90.5)	62.7 ± 12.6 (34–87)	0.005 **
PA (°)	5.3 ± 0.62 (3.6–6.6)	4.45 ±0.94 (2.6–6.5)	<0.001 **

* X^2^ test; ** *t*-test for independent variables.

**Table 4:** BIA performance in detection of sarcopenia using AUROC analysis with the L3-SMI as the reference method.

	Area (SE)	95% CI	*p*	Sp (%)	Se (%)	Cut-Off
SMM (kg)	0.679 (0.052)	0.577–0.781	0.002	62	66	36.5
SMI (kg/m^2^)	0.737 (0.048)	0.643–0.831	<0.001	66	73	11.1
FFM (kg)	0.646 (0.053)	0.542–0.750	0.010	58	59	66.8
TBW (kg)	0.690 (0.051)	0.590–0.790	0.001	60	64	48.2
PA (°)	0.762 (0.048)	0.669–0.855	<0.001	60	79	5.05

SE: standard error; Sp: specificity; Se: sensitivity.

**Table 5:** Association between BIA parameters and sarcopenia (L3-SMI) and the univariate logistic regression analysis.

	Sarcopenia (L3-SMI)					
	Yes	No	Total	X^2^; *p* *	OR (95% CI)	*p* **
SMM (kg)				7.3; 0.007	3.2 (1.4–7)	0.004
≤36.5	37 (66)	19 (38)	56 (53)			
>36.5 ^†^	19 (34)	31 (62)	50 (47)			
SMI (kg/m^2^)				14.9; <0.001	5.3 (2.3–12)	<0.001
≤11.1	41 (73)	17 (34)	58			
>11.1 ^†^	15 (27)	33 (66)	48			
PA (°)				14.9; <0.001	5.5 (2.3–13)	<0.001
≤5.05	44 (79)	20 (40)	64			
>5.05 ^†^	12 (21)	30 (60)	42			
TBW (kg)				5.3; 0.021	2.7 (1.2–6)	0.012
≤48.2	36 (64)	20 (40)	56			
>48.2 ^†^	20 (36)	30 (60)	50			
FFM (kg)				2.4; 0.12		
≤66.8	33 (59)	21 (42)	54			
>66.8 ^†^	23 (41)	29 (58)	52			
	56	50				

* χ^2^ test; ** logistic regression, ^†^ reference level. OR: odds ratio.

**Table 6:** Multivariate logistic regression analysis for the occurrence of sarcopenia.

	Sarcopenia (L3-SMI)			
	Yes	No	*p*	OR (95% CI)
SMI (kg/m^2^)			0.001	4.5 (1.9–11)
≤11.1	41 (73)	17 (34)		
>11.1	15 (27)	33 (66)		
PA (°)			0.001	4.7 (1.9–11.6)
≤5.05	44 (79)	20 (40)		
>5.05	12 (21)	30 (60)		

## Data Availability

The data presented in this study are available on request from the corresponding author. The data are not publicly available due to ethical considerations.
